# Poly(ADP-Ribosyl)ation Is Required to Modulate Chromatin Changes at c-MYC Promoter during Emergence from Quiescence

**DOI:** 10.1371/journal.pone.0102575

**Published:** 2014-07-21

**Authors:** Cassandra Mostocotto, Mariarosaria Carbone, Cecilia Battistelli, Agnese Ciotti, Paolo Amati, Rossella Maione

**Affiliations:** 1 Department of Cellular Biotechnologies and Hematology, Sapienza University, Rome, Italy; 2 Pasteur Institute-Fondazione Cenci Bolognetti, Rome, Italy; Tel-Aviv University, Israel

## Abstract

Poly(ADP-ribosyl)ation is a post-translational modification of various proteins and participates in the regulation of chromatin structure and transcription through complex mechanisms not completely understood. We have previously shown that PARP-1, the major family member of poly(ADP-ribose)polymerases, plays an important role in the cell cycle reactivation of resting cells by regulating the expression of Immediate Early Response Genes, such as c-MYC, c-FOS, JUNB and EGR-1. In the present work we have investigated the molecular mechanisms by which the enzyme induces c-MYC transcription upon serum stimulation of quiescent cells. We show that PARP-1 is constitutively associated *in vivo* to a *c-*MYC promoter region recognized as biologically relevant for the transcriptional regulation of the gene. Moreover, we report that serum stimulation causes the prompt accumulation of ADP-ribose polymers on the same region and that this modification is required for chromatin decondensation and for the exchange of negative for positive transcriptional regulators. Finally we provide evidence that the inhibition of PARP activity along with serum stimulation impairs c-MYC induction by preventing the proper accumulation of histone H3 phosphoacetylation, a specific chromatin mark for the activation of Immediate Early Response Genes. These findings not only suggest a novel strategy by which PARP-1 regulates the transcriptional activity of promoters but also provide new information about the complex regulation of c-MYC expression, a critical determinant of the transition from quiescence to proliferation.

## Introduction

Poly(ADP-ribose)polymerases, also termed ADP-ribosyltransferases with diphtheria toxin homology (ARTDs) according to a new nomenclature [1], catalyze the polymerization of ADP-ribose units from NAD^+^ on acceptor proteins, leading to the formation of linear or branched polymers of ADP-ribose. Poly(ADP-ribosyl)ation has been implicated in several distinct processes regulating chromatin structure and transcriptional activity, both at global and gene-specific levels [2]. Poly(ADP-ribose)polymerase-1 (PARP-1), an abundant and ubiquitous enzyme, is the best characterized member of a family of 17 proteins [3]. Genome-wide analysis of PARP-1 binding in tumor cells showed that the protein localizes to the promoters of almost all actively transcribed genes [4], which suggests that it plays a role in promoting the formation of chromatin structures that are permissive to transcriptional activity.

The mechanisms by which PARP-1 regulates transcription in different biological contexts seem to be very complex and are still object of investigation [2]. PARP-1 can directly bind to DNA and nucleosomes and can associate with transcription factors and co-regulatory complexes. Recent studies have suggested several ways by which PARP-1, through these multiple activities, may participate in regulating gene expression. Its binding to a wide range of DNA sequences or to specific structures can change the activity of enhancers and promoters [2]. Moreover PARP-1 binding to nucleosomes can promote, through histone poly(ADP-ribosyl)ation, the relaxation of the chromatin structure and the reorganization or repositioning of the nucleosomes [5], [6].

PARP-1 affects chromatin function also by modulating the activity and localization of DNA-methyltransferase 1 (DNMT1) [7] and histone-modifying enzymes [2]. For example, it has been shown that poly(ADP-ribosyl)ation blocks the chromatin binding of KDM5B, a histone lysine demethylase acting on trimethyl lysine 4 of histone H3, and inhibits its activity [8]. Moreover, through its ability to interact with and to modify co-repressor and co-activator complexes, PARP-1 can function as an exchange factor that promotes the switch from gene repression to gene activation in response to certain signalling pathways [9], [10].

The enzyme activity is induced by several different signals, including binding to damaged DNA or to particular DNA structures, interaction with other proteins, heat shock and post-translational modifications brought about by signal transduction mediators [2]. In this regard, PARP-1 activity has been shown to be induced downstream of the ERK cascade signaling [11]. We have reported that PARP-1 is promptly activated upon mitogen stimulation of quiescent cells [12]. Moreover, by using different experimental settings of resting fibroblasts and peripheral blood lymphocytes, we have demonstrated that the induction of PARP activity is required for the accumulation of Immediate Early Gene (IEG) products, such as c-MYC, c-FOS, JUNB and EGR-1 [12]. These findings suggested a general role for PARP-1 in linking extracellular signalling with the induction of the transcriptional program involved in the G0-G1 transition of quiescent cells. IEGs are defined as being rapidly and transiently induced by extracellular stimuli, in the absence of newly synthesized proteins. The molecular mechanisms underlying their transcriptional up-regulation involve several types of post-translational modifications of transcription factors, co-regulators and nucleosomal proteins [13]. Acetylation, phosphorylation and phosphoacetylation of histone tails have been associated with chromatin remodeling at regulatory elements of IEGs, during the early response to mitogen stimulation [13]–[15]. However, the signaling pathways and the regulatory mechanisms involved in this kind of response appear to be very complex and have not been completely elucidated. In the present work we have investigated the role of PARP activity in the chromatin modifications occurring at c-MYC promoter during growth factor-stimulation of quiescent cells. We report that PARP-1 is constitutively bound to functionally relevant sequences of the promoter and that its enzymatic product accumulates on the same region after stimulation. Moreover we show that inhibition of PARP activity prevents several of the promoter changes involved in the serum-dependent induction of c-MYC, such as the chromatin relaxation, the exchange of transcription factors and the accumulation of phosphoacetylated histone H3.

## Materials and Methods

### Cell cultures, cell cycle synchronization

Human primary foreskin fibroblasts (FB1329) [16] kindly provided by Dr. Crescenzi (Istituto Superiore di Sanità, Rome), and mouse CH310T1/2 fibroblasts were grown in DMEM (Life Technologies) supplemented with 10% FBS (Life Technologies), 100 U/ml of penicillin, and 100 µg/ml of streptomycin (EuroClone). Cells were made quiescent by serum deprivation for 72 h. 10% FBS was then added to the medium to induce cell cycle re-entry and cells were collected at different times. The inhibitor of PARP activity PJ-34 (Sigma) was dissolved in water before being added to the cell culture medium at the final concentration of 20 µM in concomitance with serum-stimulation. Anisomycin (Sigma), dissolved in DMSO, was added 30 minutes before serum-stimulation at the final concentration of 50 nM. TSA (Sigma), dissolved in ethanol, was added 3 hours before serum-stimulation and used at the final concentration of 1.6 µM.

### siRNA transfections

FB1329 human fibroblasts were made quiescent by serum starvation for 36 hours and then transfected with Lipofectamine 2000 (Life Technologies) according to the manufacturer's instructions. siRNA (purchased from Eurofin MWG Operon) were used at the final concentration of 100 nM. The siRNA oligonucleotide sequences specific for human PARP-1 (Accession number NM_001618.3) [17] were:

5′- AAG CCT CCG CTC CTG AAC AAT-3′ (nucleotides 2413-2433);

5′-AAG ATA GAG CGT GAA GGC GAA-3′ (nucleotides 2671–2691) used for the experiment reported in Figure S1;

The oligonucleotide sequence targeting GFP used as a control was:

5′- GGC UAC GUC CAG GAG CGC ACC-3′. 72 hours post transfection, cells were collected or reactivated by serum addition.

### Generation of stably knocked- down cell lines

The stably knocked-down cell line for PARP-1 was obtained by using a MicroRNA-30-adapted shRNAmir retroviral vector (Open Biosystems). Briefly a 97mer containing the miR-30 back-bone plus sense and antisense sequence targeting nucleotides 1750–1771 of mouse PARP-1 (NM_007415.2) was cloned into the MSCV-LTRmiR30-PIG (LMP) according to the manufacturer's instruction. Production of empty (Control) and shPARP-1-expressing retroviruses were performed as described previously [18]. CH310T1/2 murine fibroblasts were infected and retroviral integration was selected with puromycin. The selected stable cell lines were finally assessed for the levels of PARP-1 expression and ADP-ribose polymers. The 97mer sequence is:

5′-TGCTGTTGACAGTGAGCGCAGGGACGAACTCCTATTACAATAGTGAAGCCACAGAT

GTATTGTAATAGGAGTTCGTCCCTTTGCCTACTGCCTCGGA-3′

### Western blots

Total proteins were extracted using lysis buffer [60 mM Tris-Cl pH 6.8, 2% SDS, 10% glycerol, 5% β-mercaptoethanol], resolved by SDS-PAGE and transferred to a nitrocellulose membrane. The immunodetection was performed using anti-PARP1 (α-PARP1, sc-7150; Santa Cruz Biotechnology) or anti-TUBULIN (α-TUB, sc-8035; Santa Cruz Biotechnology) antibodies. After antibody binding, the filters were incubated with HPR-conjugated secondary antibodies (BioRad), and the blots were developed using SuperSignal West Pico kit (Pierce Chemical). The relative protein amounts were quantified with ImageJ software.

### RNA extraction and real-time PCR

Total cellular RNA from quiescent and reactivated cells was isolated and purified by High Pure RNA isolation kit (Roche) according to the manufacturer's instructions. 1 µg of total RNA was reverse-transcribed using iScript cDNA synthesis kit (BioRad). Quantitative real-time PCR (qPCR) amplifications were carried out in triplicate for each sample with 1 µl of diluted (1∶6) total cDNA using sybr green GoTaq qPCR MasterMix (Promega). Data were collected and analyzed by comparative cycle threshold method. Standard curves were done to test the efficiency of each primer pair, which sequences are reported in Table S1, and the relative amounts of transcripts of the tested genes were normalized respect to the housekeeping gene TBP.

### ChIP assays

ChIP experiments were carried out as previously described [19]. Chromatin samples were obtained from quiescent and serum-stimulated cells. For each condition, a chromatin amount corresponding to 150 µg of DNA was incubated with a specific antibody [anti-PARP-1 (α -PARP-1; ALX-210-302; Enzo Life Science), anti-Poly(ADP-ribose) (α-PAR, 4335; Trevigen), anti-CTCF (α-CTCF, 07–729; Millipore), anti-SP1 (α-SP1, sc-59; Santa Cruz Biotechnology)] or Normal Rabbit or Mouse IgG (12–370; 12–371 Merck Millipore). Immunocomplexes were purified with Protein A Sepharose (GE HealthCare) saturated with bovine serum albumin and salmon sperm. The supernatant of the IgG control was taken as Input sample. After extensive washing, pellets were dissolved in 300 µl Elution buffer and treated with RNaseA (Sigma). ChIP-enriched DNA and Input DNA, were purified by phenol-chloroform extraction and isopropanol precipitation. Purified DNA samples were analyzed by PCR or qPCR using the specific primer pairs reported in Table S2.

### DNAseI accessibility assays

For the accessibility assays approximately 4×10^6^ cells per sample were rinsed with phosphate-buffered saline (PBS) and then with ice-cold CSK buffer (10 mM HEPES [pH 7.4], 300 mM sucrose, 100 mM NaCl, 3 mM MgCl_2_). Cells were scraped from the plates, pelleted by centrifugation at 1500 rpm, and lysed in CSK-Triton buffer (CSK buffer containing 0.5% Triton X-100 supplemented with protease inhibitors) for 10 min on ice. Nuclei were collected after centrifugation at 3000 rpm for 5 min at 4°C and dissolved in 150 µL CSK-Triton buffer additioned with 50 mM MgCl_2_, divided into three aliquots and treated with different amount of DNaseI (0U, 5U and 10U) for 4 min on ice. Then the reactions were stopped by diluting samples with Proteinase K Buffer (10 mM Tris HC [pH 7.8], 1 mM EDTA [pH 7.4], 0.5% SDS, 200 ng/ml Proteinase K). Total DNA was recovered by phenolic extraction. Purified DNA samples were analyzed by PCR using primer pairs specific for c-MYC, Rhodopsin (RHO) and GAPDH promoters. RHO promoter was chosen as a reference since it was virtually not digested by DNAseI in our conditions. The PCR products were detected on agarose gel and quantified by ImageJ software.

### ChIP assays for histone modification

ChIP experiments for histone modifications were performed using Magna ChIP Protein A Magnetic Beads (Millipore) according to the manufacturer's instructions and using chromatin amounts corresponding to 50 µg of DNA. The antibodies used were: anti-Acetyl-Histone H3 (α-AcH3, 06-599; Millipore), anti-trimethyl-Histone H3 (Lys4) (α-H3K4me3, 07-473; Millipore), Anti-Histone H3 (acetyl K9, phospho S10) (α-pAcH3, ab12181; Abcam) and Normal Rabbit IgG (12–370; Millipore). Pulled-down DNA was analyzed in triplicate by qPCR and the values were normalized respect to GAPDH promoter (used as control region). Each experiment was repeated at least twice and a representative result is shown. The negative control values (not shown) were lower than the 10% of the immunoprecipitated samples.

### Statistical analysis

All the results plotted were reported as mean value ± the Standard Deviation (SD). Results obtained by qPCR were analyzed by CFX manager software (Bio-Rad) which retains the propagation of the errors between the values. Experiments with a statistical significance (i.e., qPCR, DNAseI-PCR) were conducted a minimum of three times with independent biological replicates. The significance of differences between specific and control samples was determined using a two tails paired Student's t test. The T Test was performed using the Excel software. Differences were considered to be statistically significant at P<0.05 (*) and P<0.01 (**).

## Results

### PARP-1 is required for normal accumulation of c-MYC mRNA

**Figure 1:**
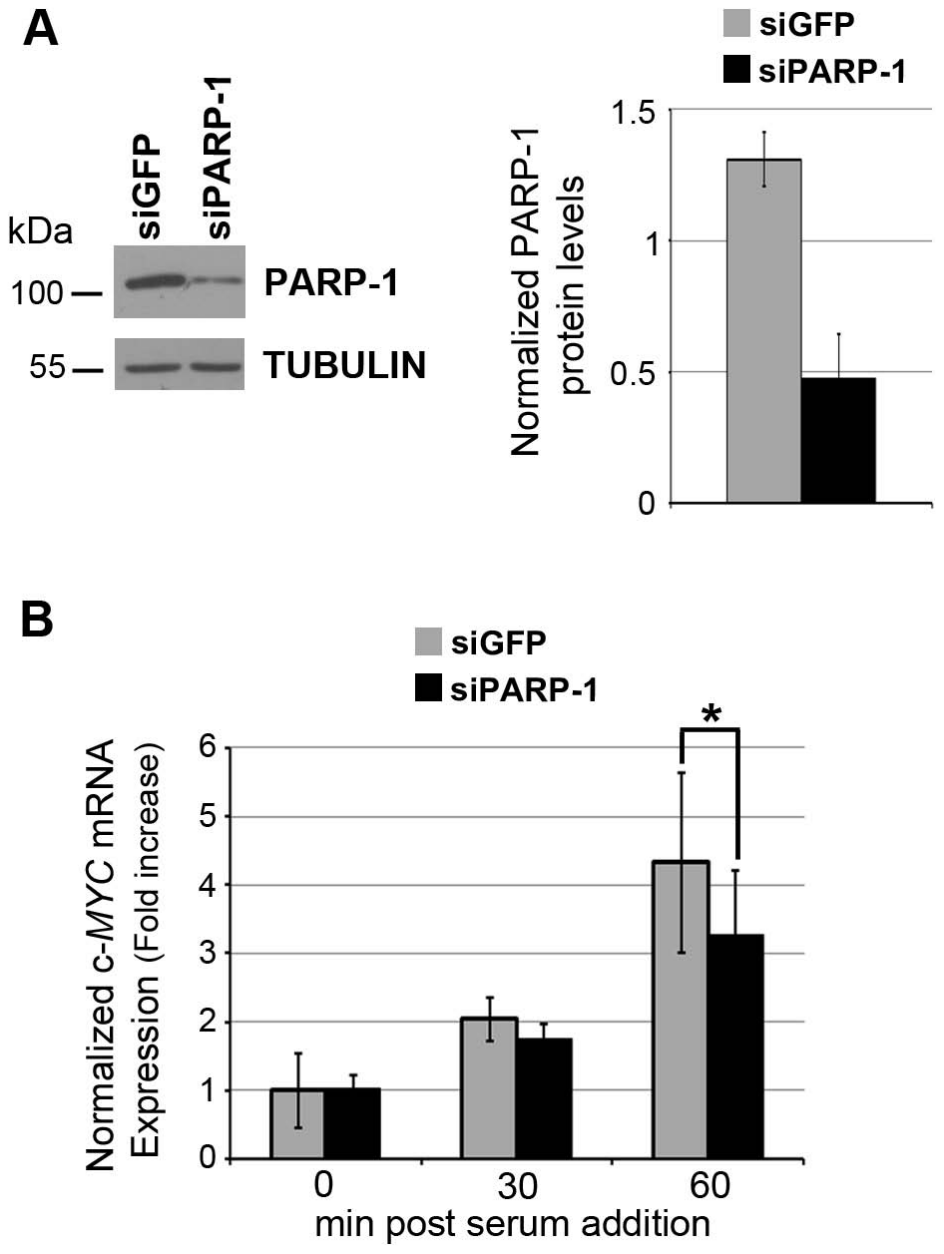
PARP-1 is required for the normal accumulation of c-MYC mRNA upon serum stimulation. A, PARP-1 levels assessed by western blot in quiescent FB1329 fibroblasts transfected with the control (siGFP) or the specific (siPARP-1) siRNAs. TUBULIN was used as a loading control. Left panel shows the results of a representative experiment; right panel shows the averages and the Standard Deviations (SD) of densitometric values of PARP-1 signals normalized respect to TUBULIN, derived from three independent experiments. B, c-MYC expression assayed by RT-qPCR in quiescent (0) and serum-stimulated (for 30 or 60 minutes) siRNA-trasfected fibroblasts. c-MYC expression levels were normalized relatively to TBP expression and reported as fold increase respect to the control quiescent sample. The error bars represent the Standard Deviation of three biological replicates. Statistical significance: *: P<0.05 (Student's t test).

The regulation of c-MYC gene expression is very complex and occurs at different levels ranging from transcriptional to post-translational control [20], [21]. We have previously shown that PARP activity is required for the accumulation of c-FOS and c-MYC mRNAs after serum stimulation of quiescent cells [12]. On the basis of this finding we first wanted to confirm that PARP-1 is the family member involved in the induction of c-MYC transcription. To this end, we performed the knockdown of PARP-1 in quiescent cells through a siRNA approach. FB1329 human fibroblasts were made quiescent by serum starvation and then transfected with a siRNA specific for PARP-1 (siPARP-1) or with a control siRNA (siGFP). Seventy-two hours post transfection cells were collected to check the efficiency of silencing or stimulated with serum to analyze c-MYC expression. Western blot analysis confirmed the decrease of PARP-1 protein levels (Figure 1A). The relative amounts of c-MYC mRNA were measured by RT-qPCR, 30 and 60 minutes after serum stimulation of transfected cells (Figure 1B). Remarkably, a significant reduction of the gene up-regulation was observed in PARP-1-depleted compared to control cells. A similar outcome was observed by using a different siRNA for PARP-1 (Figure S1), thus excluding the possibility of off-target effects. These results confirmed that PARP-1 is required for the proper induction of c-MYC expression during the G0-G1 transition of quiescent fibroblasts and suggested that this regulation occurs at the transcriptional level.

### PARP-1 binds to c-MYC promoter

**Figure 2:**
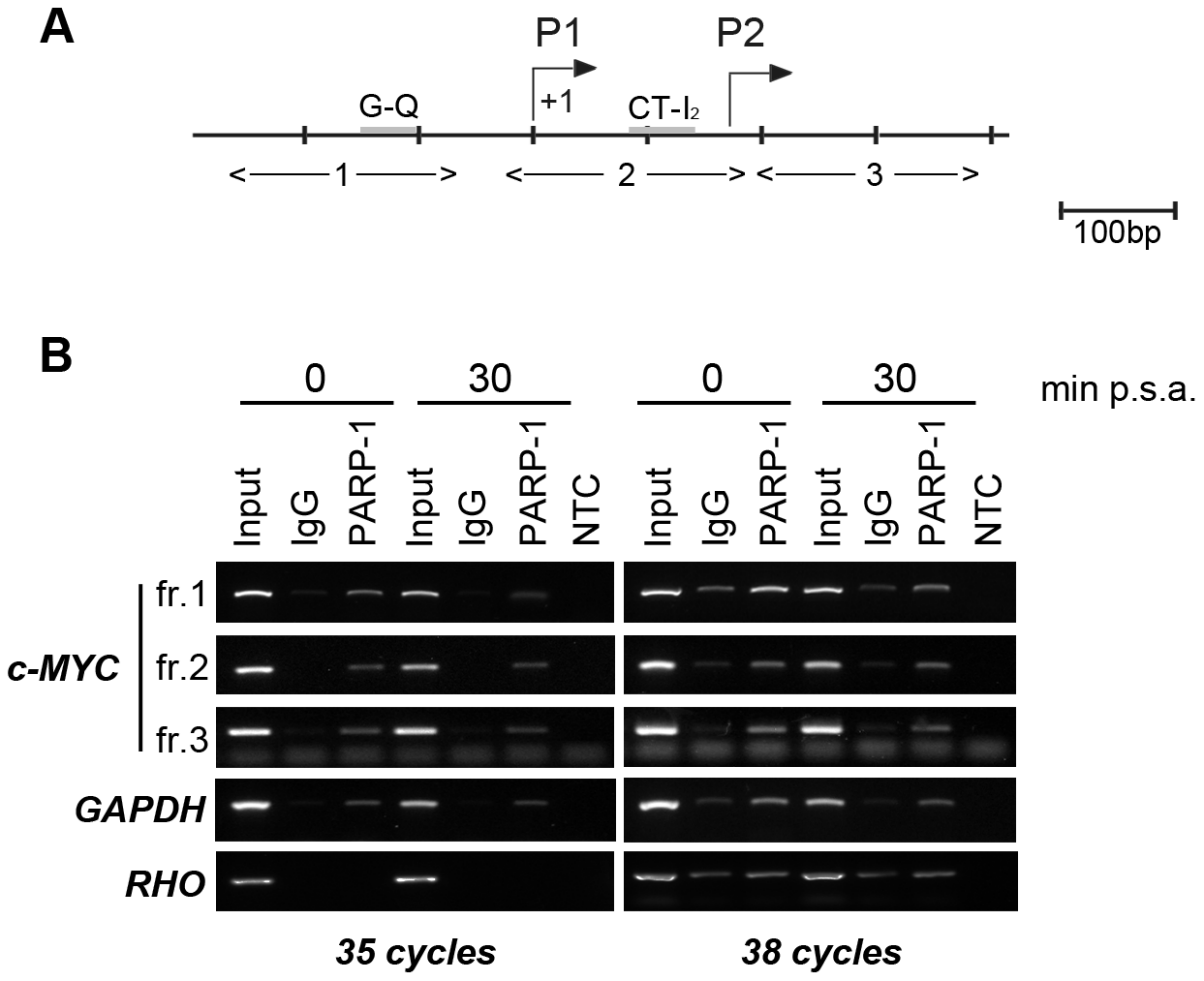
PARP-1 binds *in vivo* to c-MYC promoter. A, Schematic representation of the *c-MYC* promoter region (adapted from [21]). The transcriptional start sites P1 and P2, the Guanine-quadruplex forming element (G–Q), the consensus sequence CCCTCCCC (CT-I_2_) are shown. The fragments analyzed in B are indicated below the scheme. B, ChIP analysis of PARP-1 binding to c-MYC promoter. The assays were carried out in quiescent (0) and in 30 minutes serum-stimulated cells, using an antibody specific for PARP-1 or a normal rabbit IgG as a negative control of the immuno-precipitation. Diluted Input and immuno-precipitated samples were subjected to PCR amplification of the indicated fragments of c-MYC (fr.1, fr.2 and fr.3) or GAPDH promoter regions. RHO promoter was used as a negative control of PARP-1 binding. Numbers above the panels indicate the minutes post serum addition (min p.s.a.). Numbers below the panels indicate the cycles of PCR amplification. The results shown are representative of multiple independent experiments.

A key function in the transcriptional up-regulation of c-MYC is played by the promoter region, which has been demonstrated to be the target of a plethora of signaling pathways and transcription factors. c-MYC transcription starts from two major promoters, P1 and P2, with a predominance of P2-initiated transcripts [21]. Several structural and functional elements have been identified in the c-MYC promoter region (outlined in Figure 2A). For example, a GC-rich sequence located upstream of P1 transcriptional start site has the ability to form a G-quadruplex structure (GQ) which represses c-MYC transcription [22]. A further element, named CT-I_2_/ME1a1 and located between P1 and P2 transcriptional start sites, is required for the maintenance of an open chromatin configuration at the promoter region and plays a central role in the regulation of the activity of both promoters [23]. Moreover, at least two sites of RNA polymerase II-pausing have been identified in the promoter region: the first between P1 and P2; the second immediately downstream of P2 transcriptional start site ([21] and references therein).

To determine the role of PARP-1 in c-MYC induction, we first explored the enzyme occupancy of the promoter region, by performing ChIP assays. To this end we designed three pairs of primers useful for amplifying three fragments which cover, respectively, the GQ element (fragment 1), the region comprised between P1 and P2 transcriptional start sites (fragment 2) and the region downstream of P2 promoter (fragment 3). Chromatin samples were collected from quiescent or serum-stimulated cells and immunoprecipitated with a PARP-1-specific antibody. As reported in Figure 2B, PCR analysis of ChIP samples revealed that PARP-1 was similarly associated to all the three fragments examined, in both quiescent and serum-stimulated cells. The enzyme was also associated to the GAPDH housekeeping promoter, used as a positive control, but not to the permanently inactive rhodopsin promoter (RHO). The observation that PARP-1binds *in vivo* to the c-MYC promoter region, gave support to our hypothesis that the enzyme is involved in c-MYC induction by acting at the promoter level.

### Poly(ADP-ribosyl)ation modulates the chromatin status of c-MYC promoter

In light of our previous finding that PARP enzymatic activity increases after serum stimulation and is necessary for the induction of IEG expression [12], we asked if cell cycle re-entry was associated with changes in the poly(ADP-ribosyl)ation of c-MYC promoter.

**Figure 3:**
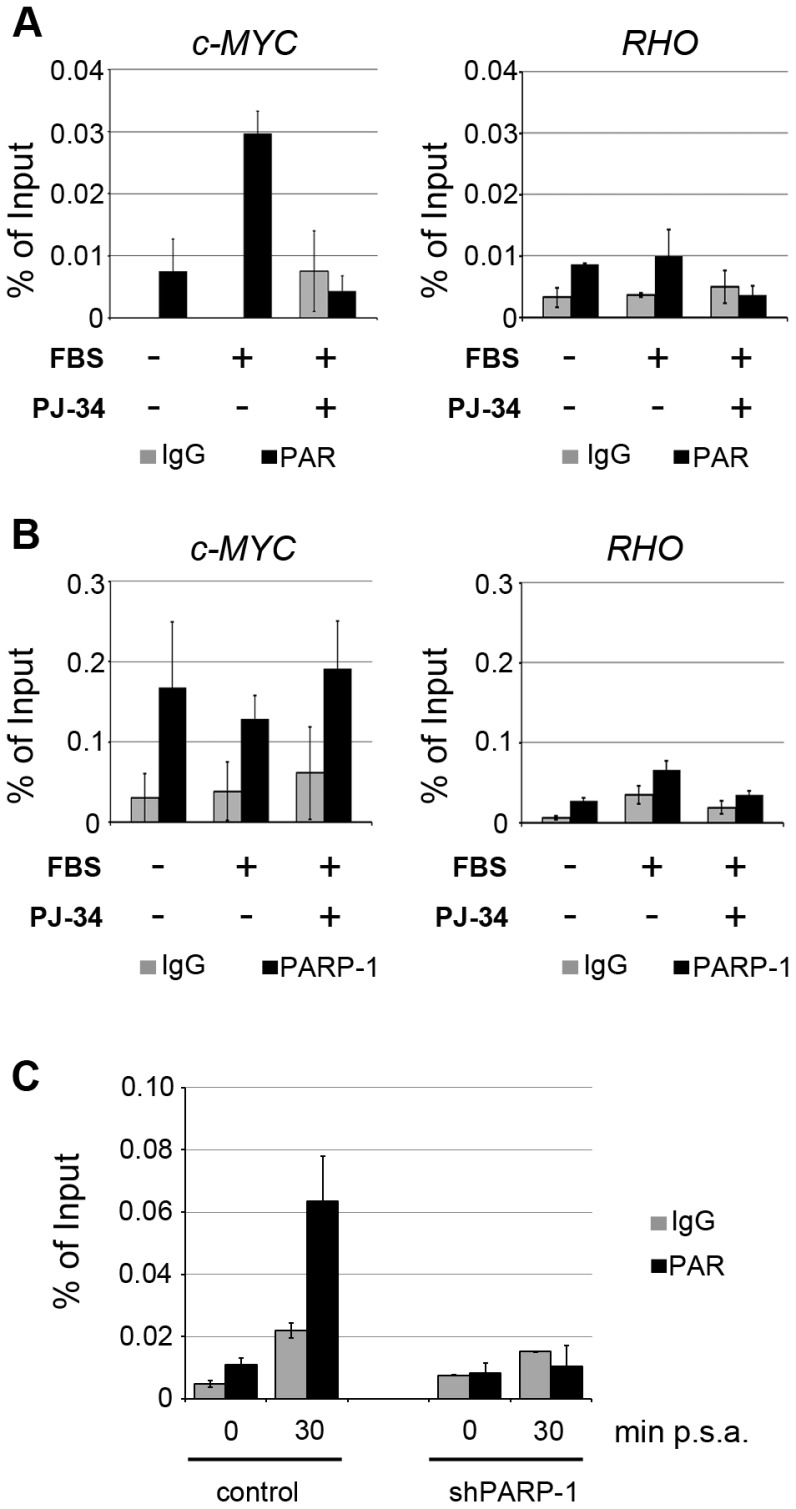
Poly(ADP-ribosyl)ation of c-MYC promoter is mediated by PARP-1. A, Chromatin poly(ADP-ribosyl)ation at c-MYC promoter investigated by ChIP assays. Chromatin samples from quiescent cells (-FBS) or from cells stimulated for 30 minutes (+FBS) in the presence or absence of the PARP inhibitor PJ-34, were immuno-precipitated with an antibody specific for poly(ADP-ribose) (PAR) or a normal mouse IgG. Input and immuno-precipitated samples were subjected to qPCR with primers specific for the fragment including both fr.2 and fr.3 (fr.2+3) of c-MYC promoter region (c-MYC). The RHO promoter was used as negative control of the binding. The results were plotted as % of Input and are representative of two independent experiments. The error bars represent the SD of three technical replicates. B, PARP-1 binding to c-MYC promoter assessed by ChIP. Chromatin samples were obtained as described in A and immuno-precipitated with an antibody specific for PARP-1 or a normal rabbit IgG. Quantitative PCR reactions were performed using the same primers as in A. Results were reported as mean ± SD of two independent experiments. C, Poly(ADP-ribosyl)ation of mouse c-MYC promoter investigated by ChIP assays in mouse fibroblasts stably knocked-down for PARP-1. Chromatin samples from quiescent cells (0) or from cells stimulated for 30 minutes, were immuno-precipitated with an antibody specific for poly(ADP-ribose) (PAR) or a normal mouse IgG. Input and immuno-precipitated samples were subjected to qPCR. The results were plotted as % of Input. Results shown are representative of two independent experiments. The error bars represent the SD of three technical replicates. min p.s.a means minutes post serum addition.

For this purpose, we performed ChIP experiments using a specific antibody recognizing poly(ADP-ribose). Quiescent and serum-stimulated cells, treated or not with the PARP inhibitor PJ-34, were collected for ChIP assays. The presence of the c-MYC promoter region in the poly(ADP-ribosyl)ated chromatin fraction was assessed by qPCR. The results, reported in Figure 3A, showed that the analyzed region becomes enriched for poly(ADP-ribose) after serum stimulation of quiescent cells. The absence of a significant PCR signal in PJ-34-treated cells not only confirmed the specificity of the ChIP assays but also supported the idea that the effects of PARP inhibition, that we previously observed on c-MYC activation [12], likely involve the failed poly(ADP-ribosyl)ation of the promoter. Importantly, the very low levels of poly(ADP-ribosyl)ation of RHO promoter correlate with the absence of PARP-1 binding. ChIP-qPCR assays for PARP-1, in addition to confirm the results reported in Figure 2B, also indicated that PARP-1 remains associated to the c-MYC promoter regardless of the enzyme activity (Figure 3B). These findings suggest that the observed poly(ADP-ribosyl)ation is mediated by promoter-bound PARP-1. To support this hypothesis we analyzed the effects of PARP-1 knock-down on the accumulation of ADP-ribose polymers on c-MYC promoter. Due to the difficulty in obtaining large populations of FB1329 fibroblasts, as is required for ChIP assays, we addressed this issue in mouse fibroblast cell lines, that we had previously demonstrated to require PARP activity for up-regulating c-myc during cell cycle re-entry [12]. To this end, we generated cell lines stably knocked-down for PARP-1 (Figure S2). ChIP assays were performed in quiescent and reactivated cells with antibodies recognizing poly(ADP-ribose) or normal mouse IgG. qPCR analysis revealed that in the absence of the enzyme, ADP-ribose polymers did not accumulate on the promoter region after serum addition (Figure 3C). Thus the family member PARP-1 plays a pivotal role in the induced poly(ADP-ribosyl)ation of c-MYC promoter during the emergence of the cells from the quiescence.

The c-MYC regulatory region contains a number of DNaseI hypersensitive sites, indicative of an accessible chromatin conformation, some of which are strictly associated with the activation of the gene transcription [21].

**Figure 4:**
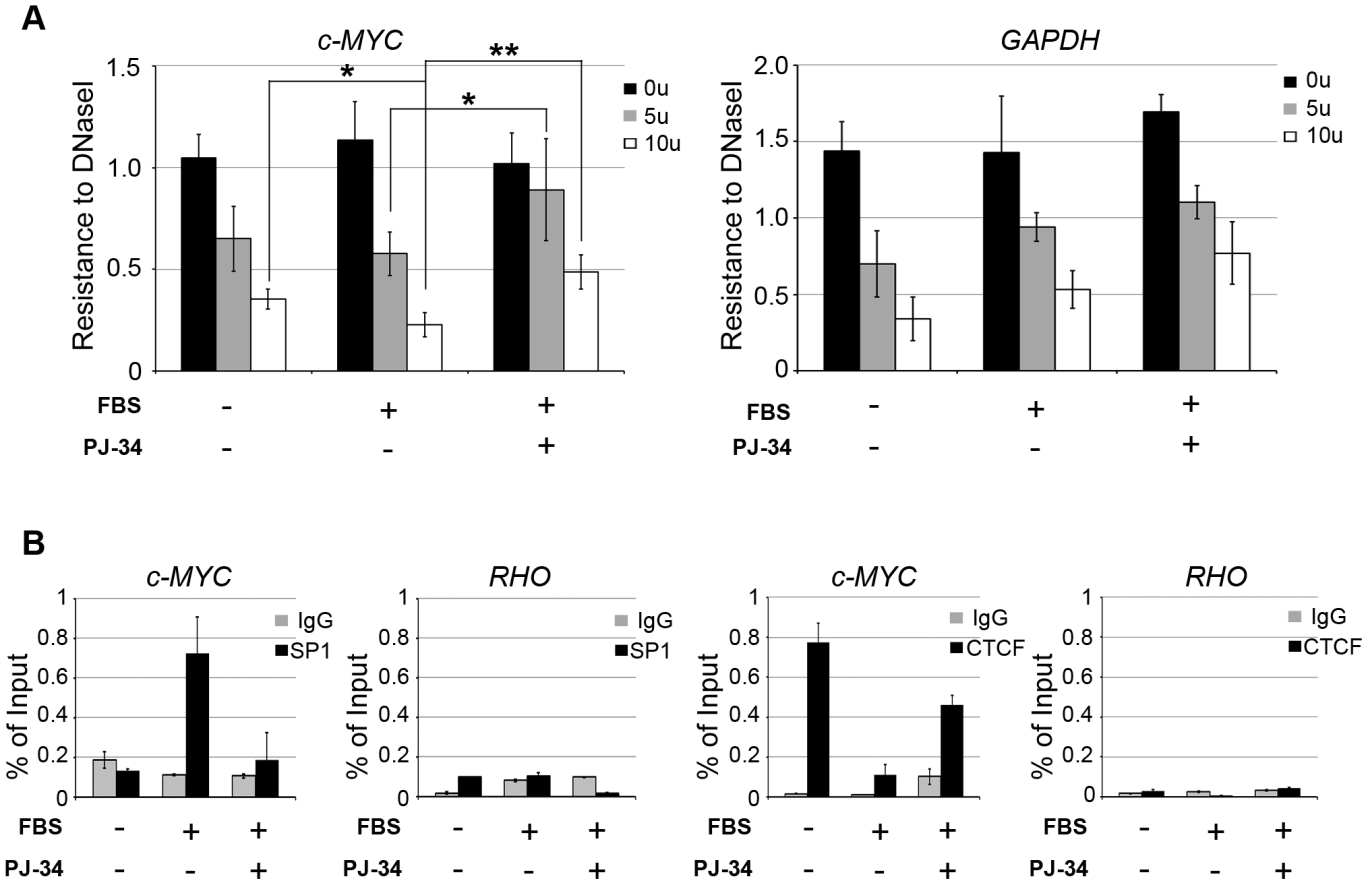
PARP activity promotes an active chromatin status at c-MYC promoter. A, DNAseI accessibility assays on c-MYC promoter. Quiescent (-FBS) and 30-minutes serum-stimulated (+FBS) fibroblasts were treated or not with the PARP inhibitor PJ-34. The entire nuclei were isolated and treated with 5 or 10 units of DNaseI or left untreated. After purification DNAs were analyzed by semi-quantitative PCR with a primer pair specific for the fragment 2 of c-MYC promoter. Densitometric-values obtained for c-MYC and GADPH promoters were normalized respect to those obtained for RHO promoter. Data are plotted as mean ± SD of four biological replicates. Statistical significances: *: P<0.05; **: P<0.01 (Student's t test). B, ChIP analysis of c-MYC promoter occupancy by SP1 and CTCF. Chromatin samples from cells treated as above were immuno-precipitated with antibodies specific for SP1 or CTCF or normal rabbit IgG. Input and immuno-precipitated samples were subjected to qPCR amplification with a primer pair specific for the region covering the fragment 2 and 3 (fr.2+3) of c-MYC promoter region (c-MYC) and for RHO promoter used as a negative control. The results shown are representative of two independent experiments and plotted as described in Figure 3A.

In light of the ability of poly(ADP-ribosyl)ation to alter chromatin structure [3] and on the basis of the accumulation of this modification on c-MYC promoter during cell cycle re-entry, we tested the effects of PARP inhibition on the accessibility of this region to DNAseI digestion. To this aim entire nuclei, isolated from quiescent and serum-stimulated cells, treated or not with PJ-34 were exposed to increasing concentrations of DNAseI, as described in Materials and Methods. DNA was purified, quantified and used as a template in PCR assays to assess the extent of DNAseI digestion (Figure 4A). Here we focused on the fragment 2 which has been demonstrated to be critical for regulating chromatin structure [23] and contains a well characterized DNAse I-hypersensitive site related to the transcriptional activity of the gene [24]. As expected, serum stimulation caused a detectable decrease of the DNAseI resistance of c-MYC promoter, respect to quiescent cells. This confirmed the enhanced chromatin accessibility of the region analyzed, consistent with the induction of the gene. Remarkably, cells stimulated in the presence of PJ-34 showed a higher degree of DNAseI resistance with respect to untreated control cells, similar to that observed in quiescent cells and indicative of a closed chromatin conformation (Figure 4A). In contrast, the housekeeping GAPDH gene promoter was equally digested by DNAseI independently of the presence of the PARP inhibitor or serum addition (Figure 4A). The differential effect of PJ-34 on the accessibility of the two promoters reflects the differential effect that the PARP inhibitor exerts on the mRNA levels of the two genes (see below). Thus the inhibition of PARP activity prevents the chromatin opening normally associated with the activation of c-MYC promoter. These data suggest that poly(ADP-ribosyl)ation alters the chromatin structure of the previously silent c-MYC promoter and that this change is necessary for the gene activation associated to the G0-G1 transition of resting cells.

The DNAseI hypersensitive sites represent highly accessible DNA regions that could be prone to dynamic changes in the binding of transcription factors. Thus we asked whether the observed chromatin changes were associated to changes in transcription factors binding on the c-MYC promoter. To this aim, we analyzed the occupancy of the region of interest by a couple of factors known to modulate c-MYC expression in an opposite manner. In particular, we focused on CTCF and SP1, which act respectively as a negative and a positive regulator of c-MYC expression and whose consensus sequences lie in the same region that we have observed to become poly(ADP-ribosyl)ated [25], [26]. Chromatin samples from quiescent and serum-stimulated cells treated or not with the PARP inhibitor, were analyzed by ChIP-qPCR assays for CTCF and SP1 binding. As reported in Figure 4B, the region of interest was not occupied by SP1 in quiescent cells but serum stimulation resulted in SP1 binding, according to the involvement of this transcription factor in the mitogen-dependent induction of c-MYC [27]. Interestingly, the association of SP1 with the promoter was prevented by PJ-34 treatment (i.e. in the absence of chromatin poly(ADP-ribosyl)ation). Conversely, CTCF was bound to the same region analyzed, in the quiescent state, and was released after serum stimulation, concomitantly with promoter poly(ADP-ribosyl)ation and c-MYC activation. Remarkably, when PARP activity was inhibited by PJ-34, CTCF remained associated with the promoter despite serum addition. The low binding levels to RHO promoter confirmed the specificity of the assays.

Taken together, these findings suggest that poly(ADP-ribosyl)ation changes the chromatin conformation at c-MYC promoter and is involved in the transcription factor exchange required for the gene induction (see Discussion)

### Poly(ADP-ribosyl)ation regulates c-MYC expression through histone modifications of the promoter

**Figure 5:**
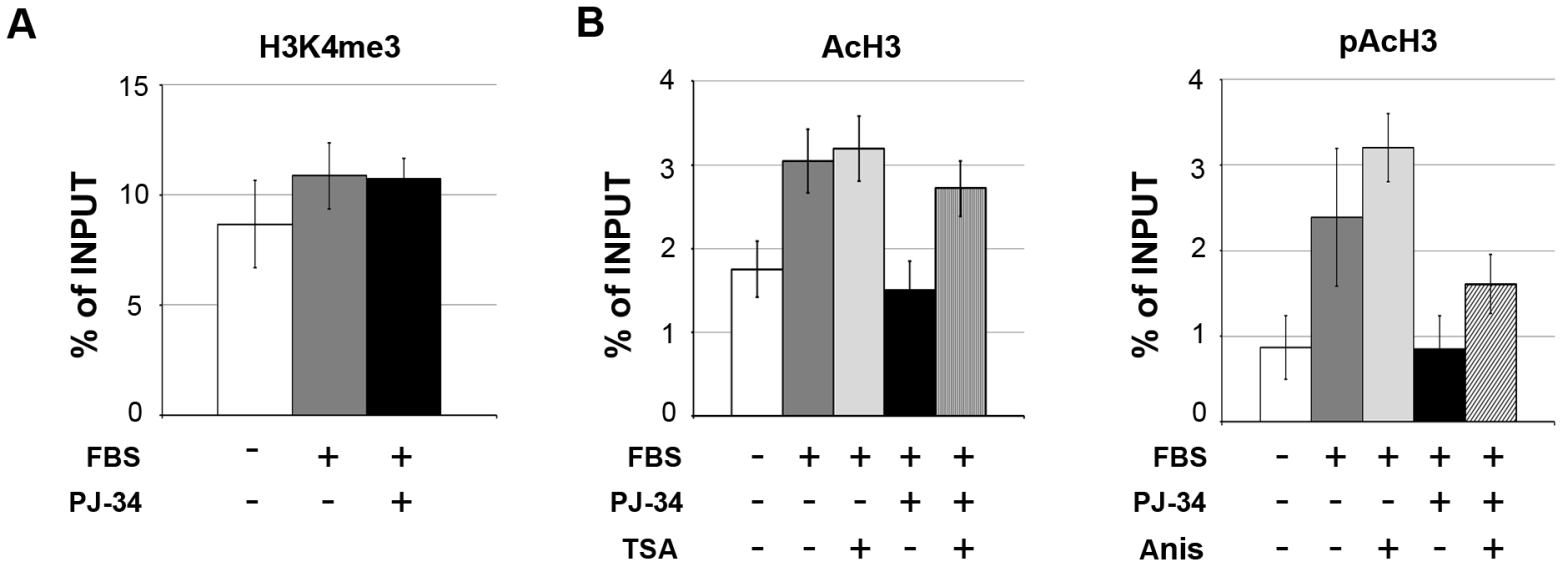
PARP-activity is required for histone modifications at c-MYC promoter. A, Histone H3K4me3 levels at c-MYC promoter assessed by ChIP-qPCR. Chromatin samples were obtained from quiescent (-FBS) or 30 minutes serum-stimulated (+FBS) fibroblasts treated or not with PJ-34, and immuno-precipitated with an antibody specific for H3K4me3. Values relative to the occupancy of c-MYC promoter (fragment fr.2+3) were normalized respect to those of GAPDH promoter and expressed as percentages of Input chromatin. The results shown are representative of two independent experiments and the error bars represent the SD of three technical replicates. B, Acetyl histone H3 (AcH3) and phosphoacetyl histone H3 (pAcH3) levels at c-MYC promoter assessed by ChIP-qPCR. Quiescent fibroblasts were pre-treated with TSA or Anisomycin (Anis) then serum-stimulated for 30 minutes in the presence or absence of PJ-34. Chromatin samples were immuno-precipitated with antibodies specific for AcH3 or pAcH3. The reported values are expressed as in A.

The reorganization of chromatin structure involves a complex and not completely understood interplay between several types of post-translational modifications of histone proteins and the activity of chromatin remodelling complexes [28]. PARP-1, in addition to directly poly(ADP-ribosyl)ate histones and chromatin-associated proteins, has been recently shown to functionally interact with other histone-modifying pathways [2]. In particular, it has been reported that PARP-1 activity is implicated in the maintenance of histone H3 lysine 4 trimethylation (H3K4me3), a mark of permissive chromatin, by inhibiting the histone demethylase KDM5B [8]. To assess whether poly(ADP-ribosyl)ation might regulate the status of c-MYC promoter by modulating H3K4me3 levels, we performed ChIP-qPCR assays in quiescent cells reactivated in the presence or absence of PARP activity. As reported in Figure 5A, the levels of H3K4me3 on this region, already clearly detectable in quiescent cells, remained substantially unmodified after serum stimulation. Moreover they also appeared insensitive to PARP inhibition. The apparent discrepancy of our results with those previously reported could be ascribed to the absence of KDM5B expression/activity in our experimental system and/or the specific involvement of different members of the KDM5 family in regulating H3K4 demethylation at c-MYC promoter. In any case our results allow us to exclude that this histone modification may play a significant role in the PARP-mediated effects of growth factors on c-MYC promoter.

**Figure 6:**
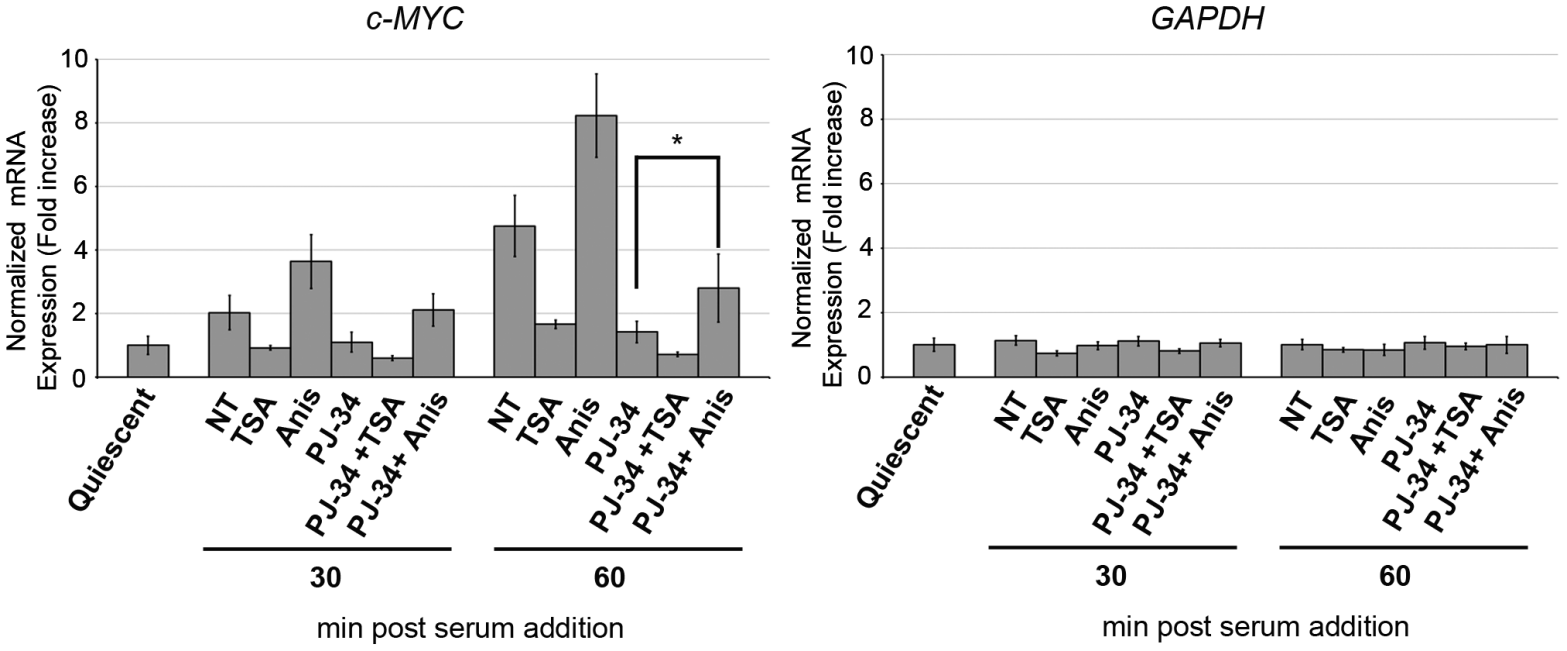
Anisomycin-stimulated phosphoacetylation restores c-MYC expression in the absence of PARP activity. c-MYC and GAPDH expression levels were assessed by RT-qPCR. Quiescent fibroblasts pre-treated with TSA or Anisomycin (Anis) or not-treated (NT), were serum-stimulated for 30 or 60 minutes in the presence or absence of PJ-34. Total RNAs were purified and the expression values obtained for c-MYC and GAPDH were normalized respect to TBP and plotted as relative to the control sample corresponding to quiescent cells. The error bars represent the SD of three biological replicates. Statistical significance: *: P<0.05.

As potential targets of PARP activity in the induction of c-MYC promoter, we considered two other marks of chromatin relaxation: acetylation and phosphoacetylation of histone H3. Acetylation of core histones is generally enriched at the 5′ of actively transcribed genes [28] and in some experimental settings it has been found to be a downstream effect of ERK signaling [13], [29], [30]. Moreover, a global increase of histone acetylation has been previously linked to PARP activation in cortical neurons and cardiomyocytes treated with signal molecules activating the ERK pathway [11]. On the other hand, phosphoacetylation of histone H3 is a double modification typically occurring at the promoters of IEGs downstream of growth factor stimulation, as demonstrated at least for c-FOS and c-JUN [31]. To verify the possible functional relationship of PARP activity with the above described modifications, we performed ChIP assays with antibodies specific for acetyl histone H3 or phosphoacetyl histone H3, in cells stimulated in the presence or absence of PJ-34. As reported in Figure 5B, increased levels of H3 acetylation accumulated on c-MYC promoter after serum stimulation of quiescent cells. This finding is in line with other observations showing the same kind of modification in concomitance with c-MYC induction [32]. In addition, the same region also became enriched with phosphopacetylated H3, revealing that this modification is linked not only with c-FOS and c-JUN [31] but also with c-MYC activation. Remarkably, the accumulation of both acetylation and phosphoacetylation of H3 was prevented by PARP inhibition, indicating that the two modifications are induced downstream of PARP activation. Similar results were obtained when the ChIP signals of the two H3 modifications were normalized to the ChIP signals of total H3 (Figure S3). This suggests that the observed changes do not reflect changes in nucleosome occupancy. In order to verify the functional relevance of the observed relationship, we analyzed the effects of increasing H3 acetylation or phosphoacetylation in the absence of PARP activity. To this end, cells stimulated in the presence of PJ-34 were co-treated with either TSA, a widely used inhibitor of histone deacetylases, or anisomycin, a strong activator of p38-MAPK-mediated phosphoacetylation [14]. The levels of H3 acetylation and phosphoacetylation on c-MYC promoter and the levels of c-MYC transcript were measured by ChIP-qPCR (Figure 5B) and RT-qPCR (Figure 6) respectively. Intriguingly, both drugs partially overcame the negative effects of PJ-34 on chromatin modifications. However, while TSA treatment did not re-establish c-MYC expression in the absence of PARP activity (see also Discussion), anisomycin treatment restored, together with promoter H3 phosphoacetylation, also the inducibility of c-MYC expression (Figure 6).

These results indicated that poly(ADP-ribosyl)ation mediates the serum-dependent up-regulation of c-MYC through a mechanism involving the modulation of other histone modifications, in particular H3 phosphoacetylation.

## Discussion

We have previously demonstrated, by means of PARP inhibition and PARP-1 knock-down, that the enzyme activation is required for resting cells to emerge from quiescence. This function involves the ability of PARP-1 to mediate the accumulation of IEG products in response to mitogen stimulation [12]. In the present work we have focused on the role of poly(ADP-ribosyl)ation in the induction of c-MYC transcription and, in particular on the functional interaction of PARP-1 with c-MYC promoter.

We show that PARP-1 occupies c-MYC promoter in both quiescent and serum-stimulated cells. One of the bound regions covers the GQ-element, a sequence able to adopt a four-stranded secondary structure named guanine (G)-quadruplexes. This structure, which is in equilibrium with the double-stranded B-DNA form, prevents the binding of transcriptional activators [33]. Interestingly, it has been previously reported that PARP-1 binds to this sequence *in vitro* [34] and that this binding accelerates the *in vitro* conversion of the c-MYC GQ structure into its B-DNA form [35]. Our ChIP assays revealed that the enzyme also binds to an adjacent region, which contains that CT-I_2_ element that has been previously recognized as biologically relevant for c-MYC transcription. This element is critical for the maintenance of an open chromatin configuration at the dual c-MYC P1/P2 promoter in episomal assays and participates in the complex regulation of initiation and elongation of P1 and P2 transcripts [23]. Notably, we observed that this region becomes rapidly poly(ADP-ribosyl)ated during emergence from quiescence. Furthermore, we observed that the same region undergoes a structural change, detectable by DNaseI accessibility assays, from a close to an open conformation. This finding is in line with previous reports showing increased nuclease sensitivity of the promoter associated with increased transcriptional activity of the gene [21]. Consistently with the promoter activation, we observed that mitogen stimulation causes the binding of SP1 and the release of CTCF from the same region. More important, we found that all these changes are prevented by inhibition of PARP activity. Taken together, these results strongly support the hypothesis that promoter-bound PARP-1, upon activation, plays a critical role in the chromatin dynamics leading to the switch on of c-MYC promoter. The proposed model is not mutually exclusive with the suggestion that the enzyme participates in c-MYC regulation by converting the GQ into the B-DNA structure [35]. Rather it is conceivable that both mechanisms can cooperate in the induction of c-MYC expression by promoter-bound PARP-1.

Concerning how PARP-1 activity may induce chromatin decondensation, transcriptional accessibility of the promoter and co-factor exchange, the simplest hypothesis would be that the enzyme directly modifies chromatin structural proteins or transcription factors. Unfortunately this case cannot be directly verified at this moment since antibodies for specific poly(ADP-ribosyl)ated proteins, to be used in ChIP assays, are not yet available. It is worth mentioning that both SP1 and CTCF have been shown to physically interact with and to be targets of PARP-1. However, as regards SP1, the poly(ADP-ribosyl)ation of this factor impairs its binding to different targets promoters [36], [37]. In addition, we have observed that PARP-1 and SP1 are efficiently co-immuno-precipitated from FB1329 fibroblast extracts regardless of quiescence, reactivation or PARP inhibition (unpublished observation). These data render unlikely a model in which SP1 is directly recruited by serum-activated PARP-1 on c-MYC promoter. Regarding CTCF, in contrast with the behaviour of many other DNA-interacting factors, poly(ADP-ribosyl)ation does not impair the binding of this factor to a variety of target sites [38]. Furthermore, as concerns our case, we did not observe any difference in the extent of CTCF poly(ADP-ribosyl)ation between quiescent and serum-stimulated cells (unpublished observation). Taken together, these findings lead us to exclude that CTCF displacement can be a consequence of the protein modification by activated PARP-1. We cannot exclude that other post-translational modifications of SP1 and CTCF and/or other regulatory factors modified by PARP-1 could mediate the observed factor exchange. However, an alternative and reasonable hypothesis is that this phenomenon is a consequence of the alterations of chromatin conformation, such as relaxation and/or nucleosome repositioning, induced by poly(ADP-ribosyl)ation.

While it is conceivable that PARP-1 causes histone poly(ADP-ribosyl)ation at c-MYC promoter, our data also revealed the existence of a functional interplay between PARP activation and other histone modifications, in the induction of c-MYC upon mitogenic signalling. In this regard, previous results showed that PARP activity is induced downstream of receptor tyrosine kinase stimulation by direct interaction with ERK2 MAP kinase [11]. Moreover, it has been well established that extracellular signal-activated pathways elicit histone modifications in concomitance with gene induction. The best studied are histone acetylation and phosphorylation [15], [39]. In particular, MAPK-mediated activation of the transcription factor Elk-1 causes the recruitment of the histone acetyl-transferase (HAT) p300/CBP leading to increased acetylation on target promoters [29], [40]. Similarly, MSK1 kinase, activated by ERK and/or p38, promotes the phosphorylation of histone H3, also known as nucleosomal response to mitogenic stimuli [14], [41]. Regarding c-fos and c-jun, it has been demonstrated that histone H3 becomes both phosphorylated (on serine 10) and acetylated (on lysine 9) upon gene activation [31]. Our results indicated for the first time that c-MYC, like c-fos and c-jun, undergoes promoter phosphoacetylation upon growth factor stimulation. What is more, the use of the PARP inhibitor allowed us to demonstrate that the proper increase of acetylation and phosphoacetylation levels of histone H3 requires PARP activity. It is worth highlighting that restoration of phosphoacetylated H3 by anisomycin treatment correlates with the restoration of c-MYC induction in the absence of poly(ADP-ribosyl)ation. We cannot exclude that anisomycin treatment may also indirectly affect c-MYC expression by targeting some transcription factor(s). However our results strongly support the functional relevance of PARP-promoted phosphoacetylation of chromatin in regulating c-MYC activation. The finding that TSA treatment, while restores H3 acetylation, does not re-establish c-MYC induction in the same conditions could simply denote that this modification is not sufficient for transcriptional activation. However, it could also reflect the complex inhibitory effects, probably involving the acetylation of other regulators, that this drug has been reported to exert on c-MYC expression in numerous studies ([21] and references therein).

**Figure 7:**
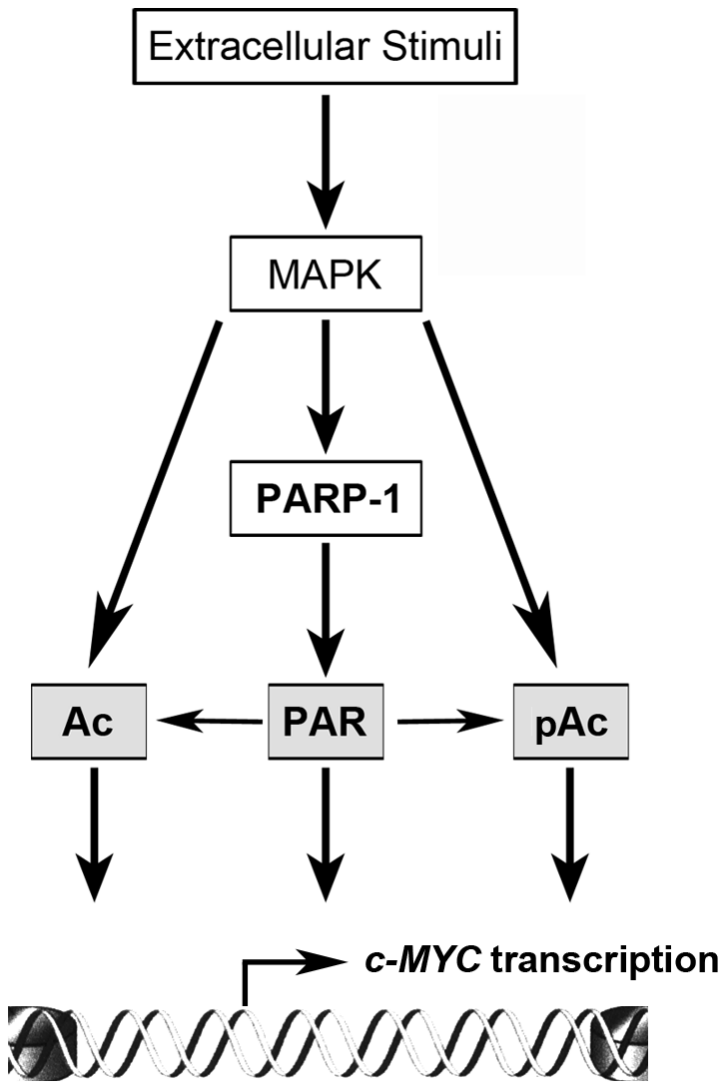
Schematic model of the functional relationship between poly(ADP-ribosyl)ation and histone modifications at c-MYC promoter. Ac: Acetylation; PAR: Poly(ADP-ribosyl)ation; pAC: Phosphoacetylation.

Our data are consistent with a model in which acetylation and phosphoacetylation at c-MYC promoter not only are directly stimulated by MAPK-activated acetylases and kinases but also are modulated by the MAPK-PARP-1-poly(ADP-ribosyl)ation pathway (Figure 7). The ability of PARP activity to influence histone acetylation has been previously suggested by a study showing that activated PARP-1 enhances pERK2-catalyzed phosphorylation of Elk-1, promoting the HAT activity of p300/CBP [11]. In that work the authors reported that nerve growth factor stimulation of cortical neurons triggers the induction of Elk-1-regulated genes, associated with a global increase of histone acetylation, in a PARP activity-dependent manner. Our results provide the first direct evidence of a functional relationship of chromatin poly(ADP-ribosyl)ation and histone acetylation within the chromatin of a specific regulatory region. Moreover, they also extend to phosphoacetylation the list of histone modifications affected by PARP activity. Although we cannot exclude that the altered histone modifications are only a consequence of the co-factor exchange induced by chromatin decompaction, the molecular mechanisms by which the enzyme influences histone acetylation and/or phosphoacetylation at c-MYC promoter are probably more complex. The role of PARP-1-induced poly(ADP-ribosyl)ation in modulating chromatin structure has been investigated in relation to the DNA damage response more extensively then in relation to transcription [42]. In this regard a current model proposes that poly(ADP-ribosyl)ated chromatin proteins function as a molecular scaffold which recruits several types of other protein complexes participating in chromatin remodeling required for DNA repair [3]. It is worth mentioning that PARP-1 has also been recognized to directly interact with a number of chromatin proteins, including acetylases and deacetylases [2] but the impact of these interactions on their transcriptional functions has not been explored enough until now. More work will be required to characterize the possible functional interaction of PARP-1 with some of the different H3 kinases and acetylases.

We would like to point out that, despite PARP-1 activity has been clearly linked to the inhibition of the histone demethylase KDM5B and to the accumulation of the permissive chromatin mark H3K4me3 [8], our results indicated that PARP-dependent activation of c-MYC promoter does not involve this histone modification. In fact, ChIP assays showed that H3K4me3 levels are already abundant in quiescent cells and do not increase after serum stimulation nor change when PARP activity is inhibited. This finding, however, is not surprising in light of the recognized role of this histone modification in marking not only active but also inactive chromatin poised for activation [43], as could be the case for c-MYC promoter.

Interestingly, we have observed the presence of PARP-1 and ADP-ribose polymers on c-FOS promoter as well (unpublished results). Moreover, a very recent work reported that PARP-1 is required for c-FOS promoter activation in HeLa cells in response to ERK MAP kinase signaling [44]. These findings suggest a picture in which the chromatin-bound enzyme would play the role of a sensor of mitogen signaling, required for allowing the prompt derepression of IEGs during the emergence from quiescence. The direct involvement of PARP-1 in the chromatin changes occurring at c-MYC promoter is currently under investigation. However, in light of the results reported in this work and on the basis of the well-established properties of the enzyme, we can reasonably hypothesize that PARP-1 is the family member responsible for the observed chromatin dynamics.

The present work suggests an additional function of PARP-1 in the regulation of chromatin organization and transcriptional activity, involving its ability to modulate the phosphoacetylation of target promoters. Moreover, it provides new information about the regulation of c-MYC promoter, a very complex and still poorly understood issue. The molecular mechanisms underlying the transcriptional induction of early response genes are of basic importance for the proper regulation of cell quiescence, a fundamental process for controlling differentiation, preserving stem cell function and preventing tumorigenesis.

## Supporting Information

Figure S1 **PARP-1 required for serum-induced accumulation of c-MYC mRNA.** A, PARP-1 levels assessed by western blot in quiescent FB1329 fibroblasts transfected with the control (siGFP) or the specific (siPARP-1) siRNAs. TUBULIN was used as a loading control. Left panel shows the results of a representative experiment; right panel shows the averages and the standard deviations (SD) of densitometric values of PARP-1 signals normalized respect to TUBULIN, derived from three independent experiments. B, c-MYC expression assayed by RT-qPCR in quiescent (0) and serum-stimulated (for 30 or 60 minutes) siRNA-transfected fibroblasts. c-MYC expression levels were normalized relatively to TBP expression and reported as fold increase respect to the control quiescent sample. The error bars represent the SD of three technical replicates.
(TIF)

Figure S2 **Poly(ADP-ribosyl)ation in cells stably knocked-down for PARP-1.** Mouse C3H10T1/2 fibroblasts expressing the PARP-1 short hairpin RNA vector (shPARP-1) or the empty vector (Control) were made quiescent by serum deprivation for 72 hrs. After serum stimulation, cells were collected and nuclei were isolated by incubation with buffer A (10 mM HEPES pH7.9; 10 mM KCl; 0.1mM EDTA; 0.1 mM EGTA; 1 mM DTT; 10% NP-40; 0.5mM PMSF). After centrifugation, nuclei were re-suspended in RIPA buffer (150 mM NaCl; 50 mM Tris HCl, pH 8; 1% NP-40; 0.5% Na-Deoxycolate; 0.1%SDS) and quantified by Lowery assay (Bio-Rad). 20 µg of nuclear extracts were resolved on SDS PAGE and transferred on a nitrocellulose membrane. The immune-detection was performed using anti-Poly(ADP-ribose) (α-PAR, 4335; Trevigen), anti-PARP1 (α-PARP1, sc-7150; Santa Cruz Biotechnology) or anti-H3 (07-690; Merck-Millipore) antibodies. The total H3 was used as a loading control. Min p.s.a. means minutes post serum addition.
(TIF)

Figure S3 **PARP-activity is required for histone modifications at** ***c-MYC*** **promoter.** Acetyl histone H3 (AcH3) and phospho-acetyl histone H3 (pAcH3) levels at *c-MYC* promoter assessed by ChIP-qPCR. Chromatin samples were obtained from quiescent (-FBS) or 30 minutes serum-stimulated (+FBS) fibroblasts treated or not with PJ-34, and immuno-precipitated with an antibody specific for AcH3, pAcH3 or total H3. Values of modified histones were normalized respect to those of total H3 in the same promoter region (fragment fr.2+3). The error bars represent the SD of three technical replicates.
(TIF)

Table S1 **Primers for RT-qPCR.**
(DOCX)

Table S2 **Primers for ChIP and DNAseI accessibility.**
(DOCX)
