# Supplementary figures and images for: Poly(ADP-Ribosyl)ation Is Required to Modulate Chromatin Changes at c-MYC Promoter during Emergence from Quiescence
Source: PLoS One. 2014 Jul 21;9(7):e102575. doi: 10.1371/journal.pone.0102575 (PMC4105440; doi:10.1371/journal.pone.0102575)

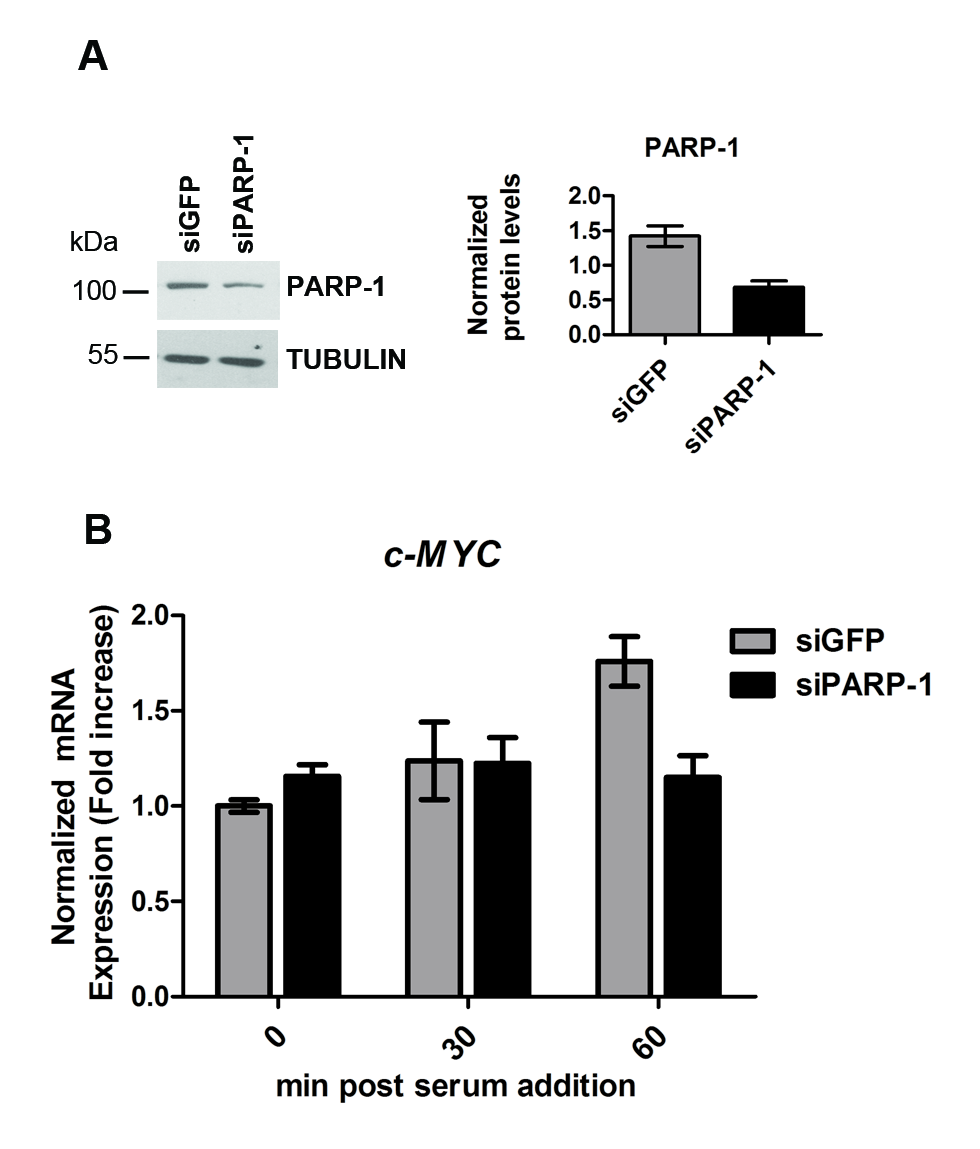

Supplement: Figure S1 — PARP-1 required for serum-induced accumulation of c-MYC mRNA. A, PARP-1 levels assessed by western blot in quiescent FB1329 fibroblasts transfected with the control (siGFP) or the specific (siPARP-1) siRNAs. TUBULIN was used as a loading control. Left panel shows the results of a representative experiment; right panel shows the averages and the standard deviations (SD) of densitometric values of PARP-1 signals normalized respect to TUBULIN, derived from three independent experiments. B, c-MYC expression assayed by RT-qPCR in quiescent (0) and serum-stimulated (for 30 or 60 minutes) siRNA-transfected fibroblasts. c-MYC expression levels were normalized relatively to TBP expression and reported as fold increase respect to the control quiescent sample. The error bars represent the SD of three technical replicates. (TIF) [file pone.0102575.s001.tif]

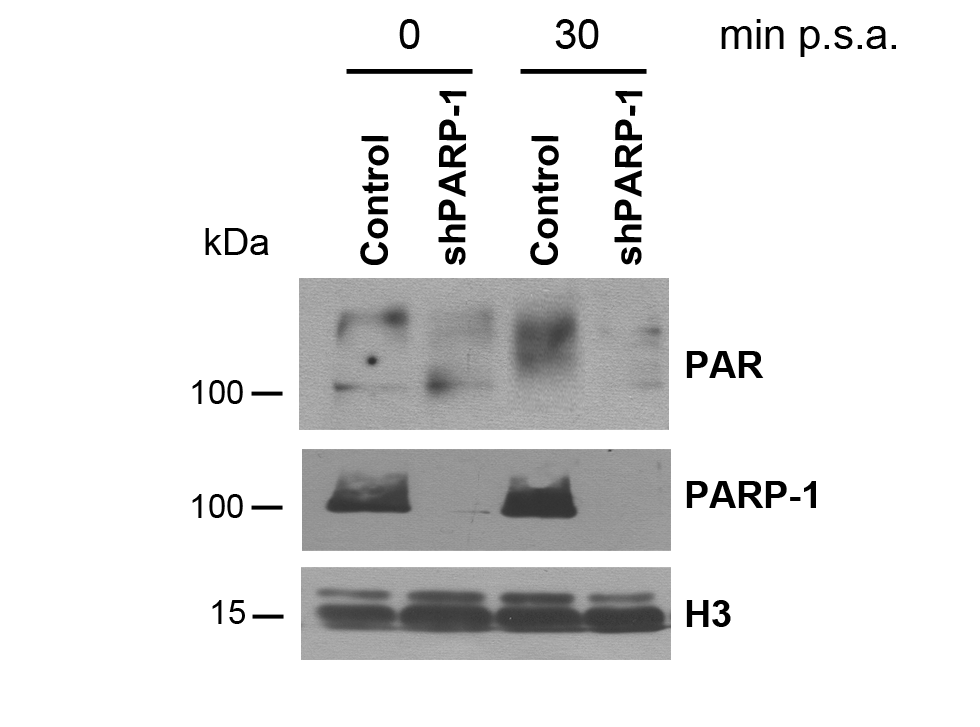

Supplement: Figure S2 — Poly(ADP-ribosyl)ation in cells stably knocked-down for PARP-1. Mouse C3H10T1/2 fibroblasts expressing the PARP-1 short hairpin RNA vector (shPARP-1) or the empty vector (Control) were made quiescent by serum deprivation for 72 hrs. After serum stimulation, cells were collected and nuclei were isolated by incubation with buffer A (10 mM HEPES pH7.9; 10 mM KCl; 0.1mM EDTA; 0.1 mM EGTA; 1 mM DTT; 10% NP-40; 0.5mM PMSF). After centrifugation, nuclei were re-suspended in RIPA buffer (150 mM NaCl; 50 mM Tris HCl, pH 8; 1% NP-40; 0.5% Na-Deoxycolate; 0.1%SDS) and quantified by Lowery assay (Bio-Rad). 20 µg of nuclear extracts were resolved on SDS PAGE and transferred on a nitrocellulose membrane. The immune-detection was performed using anti-Poly(ADP-ribose) (α-PAR, 4335; Trevigen), anti-PARP1 (α-PARP1, sc-7150; Santa Cruz Biotechnology) or anti-H3 (07-690; Merck-Millipore) antibodies. The total H3 was used as a loading control. Min p.s.a. means minutes post serum addition. (TIF) [file pone.0102575.s002.tif]

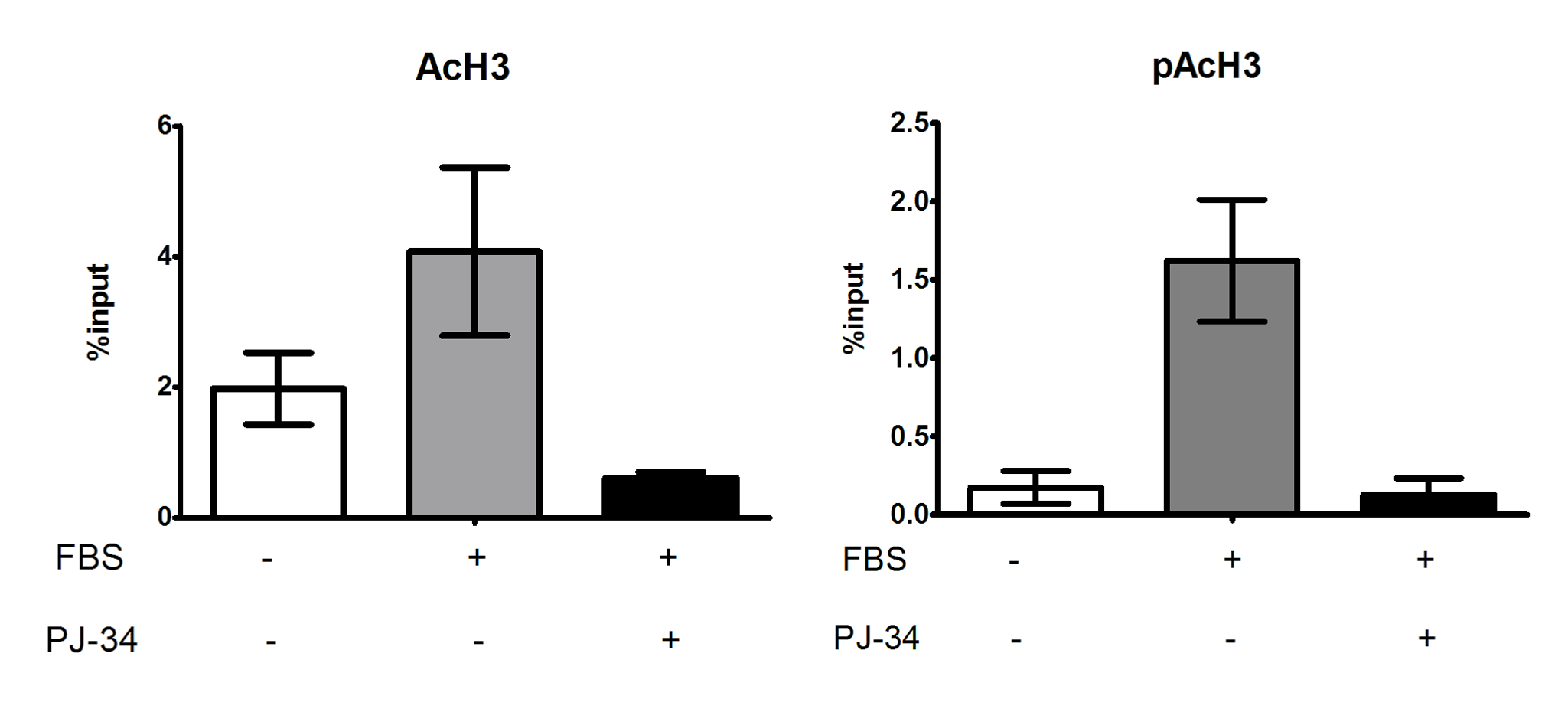

Supplement: Figure S3 — PARP-activity is required for histone modifications at c-MYC promoter. Acetyl histone H3 (AcH3) and phospho-acetyl histone H3 (pAcH3) levels at c-MYC promoter assessed by ChIP-qPCR. Chromatin samples were obtained from quiescent (-FBS) or 30 minutes serum-stimulated (+FBS) fibroblasts treated or not with PJ-34, and immuno-precipitated with an antibody specific for AcH3, pAcH3 or total H3. Values of modified histones were normalized respect to those of total H3 in the same promoter region (fragment fr.2+3). The error bars represent the SD of three technical replicates. (TIF) [file pone.0102575.s003.tif]
