# Supplementary material for: Poly(ADP-Ribosyl)ation Is Required to Modulate Chromatin Changes at c-MYC Promoter during Emergence from Quiescence
Source: PLoS One. 2014 Jul 21;9(7):e102575. doi: 10.1371/journal.pone.0102575 (PMC4105440; doi:10.1371/journal.pone.0102575)
Supplement: Table S1 — Primers for RT-qPCR. (DOCX) [file pone.0102575.s004.docx]

**Table S1: Primers for RT-qPCR**

| Accession Number | Symbol | Sequences(5’-3’) | Nucleotides |
| --- | --- | --- | --- |
| NM_003194.4 | TBP | For: GCTGGCCCATAGTGATCTTT | 100-119 |
|  |  | Rev: CTTCACACGCCAAGAAACAGT | 156-136 |
| NM_002467.4 | c-MYC | For: CGCCCTCCTACGTTGCGGTC | 782-801 |
|  |  | Rev: CGTCGTCCGGGTCGCAGATG | 934-915 |
| NM_002046.4 | GAPDH | For: GACAGTCAGCCGCATCTTCT | 119-138 |
|  |  | Rev: GCGCCCAATACGACCAAATC | 222-203 |
