# Supplementary material for: Poly(ADP-Ribosyl)ation Is Required to Modulate Chromatin Changes at c-MYC Promoter during Emergence from Quiescence
Source: PLoS One. 2014 Jul 21;9(7):e102575. doi: 10.1371/journal.pone.0102575 (PMC4105440; doi:10.1371/journal.pone.0102575)
Supplement: Table S2 — Primers for ChIP and DNAseI accessibility. (DOCX) [file pone.0102575.s005.docx]

**Table S2: Primers for ChIP and DNAseI accessibility**

| Accession Number | Gene Symbol | Sequences (5’-3’) | Amplicon |
| --- | --- | --- | --- |
| D10493.1 | c-MYC | For1 : GAGGAGCAGCAGAGAAAG | **Fr1**  2043-2284 |
|  |  | Rev1 : CGGAGATTAGCGAGAGAGGA |  |
|  |  | For2 : CTTTAAATGCGAGGGTCTGG | **Fr2**  2295-2516 |
|  |  | Rev2 : TGCCTCTCGCTGGAATTACT |  |
|  |  | For3 : GCCGGCTAGGGTGGAAGA (*) | **Fr3**  2534-2742 |
|  |  | Rev3 : CCTTGCTCGGGTGTTGTAAGTT (*) |  |
|  |  | For2’: CTCTCTCGCTAATCTCCCG (**) | **Fr2+3**  2267-2742 |
|  |  | Rev3 : CCTTGCTCGGGTGTTGTAAGTT (**) |  |
| NG_007073.2 | GAPDH | For : TCGGGGCCCACACGCTCGGTGCGTG (**) | 4801-5057 |
|  |  | Rev : GGCTGACTGTCGAACAGGAGGAGCA (**) |  |
| U16824.1 | RHO | For : TGGATCCTGAGTACCTCTCCTCCCT (**) | 5138-5358 |
|  |  | Rev : GGCTCCAGCTGGATGACTCTGGGTT (**) |  |
|  | | | |
| NM_001177354.1 | c-myc | For : GCTTGGCGGGAAAAAGAAGG (**) | 135-330 |
|  |  | Rev : AAAGCCCCTCTCACTCCAGA (**) |  |

According to the nucleotide sequence D10493.1, the major P1 promoter is localized between nucleotides 2298-2302; the major P2 promoter is localized between nucleotides 2458-2462.

According to the nucleotide sequence NM_001177354.1, the major P1 promoter is localized at nucleotide 46; the major P2 promoter is localized at nucleotide 211.

(*) Hu, H.-M. , Kanda, K., Zhang, L. and Boxer, L.M. (2007) Activation of the c-myc p1 promoter in Burkitt's lymphoma by the hs3 immunoglobulin heavy-chain gene enhancer *Leukemia* **21,** 747–753.

(**) Validated for qPCR
